# M. Ricord on the Tertiary Forms of Syphilis, and Their Treatment

**Published:** 1846-03

**Authors:** 


*M. Ricord on the Tertiary forms of Syphilis, and their treatment.*—Tertiary symptoms may be said to be essentially characterized by not being transmissible hereditarily. When syphilis has arrived at this stage, it seems to lose its specific character, and to constitute a cachexia, having a certain affinity to scrofula. I believe, it indeed, to be the most frequent cause of that malady. The constitution is deeply modified after passing through the different stages which intervene between the first manifestation of the primary sore and that of tertiary disease. The affiliation cannot always be traced, although, no doubt, it always exists. The secondary symptoms may not have been perceived, or may have been forgotten. Thus when transmitted by heredity, secondary symptoms appear within five or six months of birth, and subsequently disappear, whether treated or not. They may not be recognised by the parents; and yet tertiary symptoms supervening, the latter may appear to occur primarily.

Mercury may prevent the manifestation of tertiary symptoms, or modify them when they have appeared; but because it does not always prevent their occurrence, we are not warranted in concluding that it causes them, or that they are the result of the combined influence of mercury and syphilis, as has been asserted by many pathologists. They have been repeatedly observed on persons who have never undergone mercurial treatment—a fact which decides the question.

It is generally believed that tertiary symptoms are more serious and more difficult to cure in patients who have taken mercury than in those who have not; and among the former, more difficult to overcome, the greater the amount of mercury taken. The explanation of the fact may be easily given. The disease is most difficult to cure in such persons, because they are precisely those on whose economy syphilis has the firmest hold, and who have been, consequently, all along, the most rebellious to the action of mercury.

Tertiary symptoms are more serious than secondary, for the older syphilis is, the more formidable it becomes, and the more firmly does it establish itself in the economy which it deteriorates. Mercury, when it is not the most precious and the most efficacious of medicine, is a dangerous remedy, often, indeed, a violent poison. When it does not cure, it does not remain innocuous; on the contrary, it injures and debilitates the constitution. Now syphilis is the more formidable, the weaker the constitution on which it acts. In this sense, the abuse of mercurial preparations may be followed by deplorable results, as it places the patient in the most favourable circumstances for the development of tertiary phenomena. But although, in diminishing the quantity of fibrine in the blood, which mercury does, you weaken the constitution, you cannot, by such means alone, produce tertiary symptoms, with their peculiar characteristics. The caries which may be occasioned by carrying mercurial stomatitis to its last limits cannot be confounded with the caries of tertiary syphilis. Between the one and the other, there is the greatest possible difference.

Tertiary syphilitical symptoms rarely manifest themselves within six months of the primary infection; this is the inferior limit. As to the superior one, the longest term after which they may appear it is impossible to fix. No tertiary symptoms have been known to occur, ten, fifteen, or twenty years after infection, or even after a still longer period. It rarely happens, however, when the interval is so lengthened, that the patient has not experienced some intermediate symptom. There has nearly always, been a thorn somewhere, some local ailment, notwithstanding that the general health has been excellent. Suddenly, a tibia becomes painful, a testicle swells or melts, or an exostosis appears. A syphilitical exostosis has, however, never been known to develop itself eight, ten, or fifteen days after the manifestation of a chancre. In some rare cases, I have seen patients at the third, fourth, or fifth month, present phenomena which occupied a medium position between secondary and tertiary syphilis, and which, subsequently, became evidently tertiary; but they were exceptional cases. Tertiary accidents are principally situated in the sub-cutaneous or sub-mucous cellular tissue, in the fibrous, osseous, cartilaginous, muscular, or nervous tissues, and in organs in their locality. One of the principal characteristics of tertiary syphilis is its tendency to concentrate itself in the internal organs. One of the earliest diseases of this period is syphilitical sarcocele.

Syphilitical sarcocele has also been called syphilitical albuginitis, and justly so, as it is the white tissues that are first affected. Generally speaking, it only attacks one side, although it is not very rare to see it begin simultaneously or successively in both testicles. The attack is scarcely ever preceded by symptoms which indicate what is about to take place. When, however, this is the case, acute pains and a sensation of heaviness are experienced in the region of the loins; these pains are sometimes nocturnal. Most frequently the patient is not himself aware when the disease begins to appear, his attention being accidentally directed to its existence. The disease commences generally by the body of the testicle, on which may be felt several small indurated points, which gradually extend from one to the other, often forming, as it were, zones or circles of induration. The indurations thus continue to extend and to coalesce until the whole testicle has been invaded by the malady. The testicle has then lost its elasticity, has become coagulated and hardened. At the same time there are no symptoms indicating inflammation, and the epididymis remains soft and free. The hardened testicle may continue of normal size, or it may increase in volume until it becomes two or three times as large as natural, or even larger. The syphilitical testicle is not lobulated, nor does it present mammillary eminences. Its surface may be uneven, but the unequal surface presents a certain regularity, and never rises into protuberances. When the testicle is very voluminous, the epididymis disappears, owing to its being spread out and flattened on the superior extremity of the enlarged organ. The spermatic cord also remains healthy and free from hardness. There is no pain in the testicle, nor in its vicinity, either when the testicle is left to itself, or when pressure is exercised upon it. The weight of the enlarged organ may, however, drag down the cord, and occasion pain.

The tumour is hard, solid, without elasticity, and offers to the finger on pressure the sensation that would be produced by fibrous or cartilaginous tissues. The skin undergoes no alteration, but remains moveable, and of the natural colour. There is no febrile or functional reaction. The spermatic secretion diminishing gradually, especially if both testicles are attacked at the same time, the power of erection diminishes, and at last disappears entirely. The progress of syphilitical sarcocele is slow, essentially indolent; it may last not only several months, but several years. The tumour never suppurates, and shows no tendency to suppuration, nor does it ever go beyond the size I have mentioned. Arrived at a certain stage of its development, resolution commences, and a process sets in, the very reverse of the one I have described, either under the influence of proper treatment, or spontaneously. The testicle gradually regains its normal size, and the patient is delighted; but he soon becomes alarmed to find the diminution in size persisting until the testicle melts and disappears. As the testicle diminishes and becomes atrophied, the entire economy is altered, and the voice changes its tone.

The disease does not, however, always follow this course. There are cases in which cartilaginous, fibrous, or even osseous degeneration follow the syphilitical hypertrophy of the testicle. All the above changes may also take place in a limited point of the organ. In some exceptionable cases I have known syphilitical sarcocele to terminate by true resolution, the organ returning to its normal condition.

The disease, as I have stated, nearly always begins in the body of the testicle. I believe, however, that it may possibly commence in the epididymis in a manner analogous to that which I have described.

Can syphilitical sarcocele manifest itself in a person in whom the syphilitical diathesis has been transmitted by heredity? All that has been written on the subject of this disease has been drawn from the observation of adults who had been themselves infected, who had had chancres. It cannot, however, be denied, that syphilitical sarcocele, which is a late symptom—a tertiary one—may be the result of the hereditary transmission of syphilis, and yet only appear at the age of puberty, when the genital organs first begin to play a part in the economy. I believe, myself, that a host of testicular degenerations, which have hitherto remained unexplained, may be attributed to this disease, as, for instance, the atrophy of the testicle occurring in early life, and generally considered congenital, and many of the fibrous, cartilaginous, and osseous degenerations which we find described at every page of surgical works on the testes. All these, I firmly believe, are often only the results of syphilitical sarcocele transmitted by heredity. The same may be said of many cases of chronic orchitis, which, when not specifically treated, is often known to end in atrophy.

The causes which more immediately occasion the manifestation of syphilitical sarcocele are all those which, setting aside syphilis, are likely to irritate the testicle. The most frequent of these is blennorrhagia—a blow, a contusion, will act in the same way. The tubercular, cancerous, or scrofulous diathesis, may contribute to bring to light the syphilitical diathesis. These affections may also, as a matter of course, complicate the syphilitical disease.

Syphilitical sarcocele may be complicated by all the known diseases of the testicle and of the annexed organs, by all the forms of hernia, by varicocele, by hydrocele, by acute or chronic epididymitis, by simple orchitis, by kysts, or by the various degenerations. When, however, it exists alone, the symptoms which I have above enumerated will suffice to enable you to establish a diagnosis. If there is any doubt, a trial of the specific treatment will throw light on the question.

The prognosis will depend on the freedom from complication, and on the duration of the disease. If it is uncomplicated, and of short duration, the specific treatment of tertiary symptoms may bring the testicle to a normal state, without leaving any trace of the disease. At a more advanced epoch, it is still possible to prevent degeneration, but atrophy will often follow, which is better than retaining a hypertrophied testicle. At a still more advanced period, when hypertrophy has set in, we must endeavour to prevent degeneration, which may be all we can accomplish.

Sometimes, in mixed cases, or cases in which the symptoms of cancerous or tuberculous degeneration complicate those of syphilis, so as to render it impossible to form a correct opinion of the share which each has in the disease, the specific treatment must be resorted to as a means of elucidating the question. Unless a testicle be unquestionably cancerous or tuberculous, it should never be attacked with the knife until mercury and the iodide of potassium have been tried.—*Ibid.*

